# Sexing of chicken eggs by fluorescence and Raman spectroscopy through the shell membrane

**DOI:** 10.1371/journal.pone.0192554

**Published:** 2018-02-23

**Authors:** Roberta Galli, Grit Preusse, Christian Schnabel, Thomas Bartels, Kerstin Cramer, Maria-Elisabeth Krautwald-Junghanns, Edmund Koch, Gerald Steiner

**Affiliations:** 1 Clinical Sensoring and Monitoring, Anesthesiology and Intensive Care Medicine, Faculty of Medicine, TU Dresden, Dresden, Germany; 2 Clinic for Birds and Reptiles, Faculty of Veterinary Medicine, University of Leipzig, Leipzig, Germany; 3 Faculty of Physics, Vilnius University, Vilnius, Lithuania; Institute of Materials Science, GERMANY

## Abstract

In order to provide an alternative to day-old chick culling in the layer hatcheries, a noninvasive method for egg sexing is required at an early stage of incubation before onset of embryo sensitivity. Fluorescence and Raman spectroscopy of blood offers the potential for precise and contactless in ovo sex determination of the domestic chicken (*Gallus gallus* f. dom.) eggs already during the fourth incubation day. However, such kind of optical spectroscopy requires a window in the egg shell, is thus invasive to the embryo and leads to decreased hatching rates. Here, we show that near infrared Raman and fluorescence spectroscopy can be performed on perfused extraembryonic vessels while leaving the inner egg shell membrane intact. Sparing the shell membrane makes the measurement minimally invasive, so that the sexing procedure does not affect hatching rates. We analyze the effect of the membrane above the vessels on fluorescence signal intensity and on Raman spectrum of blood, and propose a correction method to compensate for it. After compensation, we attain a correct sexing rate above 90% by applying supervised classification of spectra. Therefore, this approach offers the best premises towards practical deployment in the hatcheries.

## Introduction

The increasing specialization of chicken lines for either egg production or meat yield performance has made the male layers worthless, so that the freshly hatched cockerels are immediately eliminated. Several approaches of chicken egg sexing have been proposed in the attempt to solve the dilemma between economy and ethics by providing an alternative to day-old chick culling in layer hatcheries.

Both noninvasive and invasive sexing methods have been explored in the recent years. Among the noninvasive ones, reflectance spectroscopy provided good sexing results at mid incubation periods [1], while hyperspectral imaging enabled reliable sexing limited to layer strains with sex-specific feather color at mid incubation periods [2]. Other totally noninvasive sexing methods based on egg shape [3] or odor [4] were not yet practically applied. Among the invasive methods that require extraction of samples, molecular sexing proved to be simple and robust in laboratory conditions [5]. Measurement of hormones in egg fluids is also a robust and established method, but it can be applied only after day 9 of incubation [6–8]. Infrared absorption spectroscopy provided the sex of unincubated eggs by analyzing the blastoderm cells [9]. However, this method could not be exploited because shell windowing of the unincubated egg strongly affects hatching rate and chick health [10,11].

We recently demonstrated that near infrared fluorescence and Raman spectroscopy (hereinafter referred to as optical spectroscopy) performed during the fourth day of incubation provides precise in ovo sexing based on differences in the composition of embryonic blood [12,13]. Although the spectroscopic analysis itself demonstrated to be totally damage-free, the method qualified nevertheless as invasive due to the need of windowing the egg shell to optically access the extraembryonic blood vessels, which led to some reduction of hatching rate (~ 10%) [12]. The measurements were performed at the pointed pole, where egg membranes are tightly adherent to the shell. Therefore, also the shell membranes were removed together with the shell by lifting of the window, directly exposing the embryo to the external environment.

It is widely accepted that the egg shell membranes, and not the shell, provide main protection against bacterial penetration [14]. The inner shell membrane, constituted by a dense mesh of interwoven fibrous proteins plus a variety of other proteins and peptides associated with protective functions [15], is a more effective barrier than the outer shell membrane [16]. In fact, only the thick mineralized shell constitutes an optical barrier to the spectroscopic measurements, while the underlying membranes are thin enough to be transparent. In this study, we show that in ovo sexing by optical spectroscopy can be performed without removing the inner shell membrane, thus avoiding negative effects on hatching rates.

## Methods

### Ethics statement

According to German animal welfare legislation, no approval was necessary to perform the experiments on embryonated chicken eggs. Egg shell opening and optical measurements were performed on embryos in early stages of development (between E3 and E4), before the neural tube has developed into a functional brain and the embryo has developed sensitivity to pain [17,18]. No experiments took place at later developmental stages. Hatched chicks were only subjected to visual observation after hatching, and then transferred to a farm. Maximal care was used in order to avoid distress to animals.

### Egg handling

Fertilized eggs of a commercial white layer strain of the domestic chicken (*Gallus gallus* f. dom.) (LSL—Lohmann Selected Leghorn) were used. They were obtained from Lohmann Tierzucht GmbH (Cuxhaven, Germany). The freshly laid eggs were inspected for any shell damage and then stored for max. 36 hours at approximately 14°C until start of the incubation. The incubation was performed in an automatic egg incubator (Favorit Olymp 192, Heka-Brutgeräte, Rietberg, Germany) at 37.8°C and a humidity of 53%, in vertical position with the blunt pole upward. A ± 45° tilting at an interval of 3 h was applied during the incubation time.

At day 3.5 (i.e., 84 h), the eggs were subjected to the measurement. The egg shells were windowed using a 30 W CO_2_ laser (Firestar v30, Synrad, Mukilteo, Washington, USA) equipped with scanning head (FH Flyer, Synrad, Mukilteo, Washington, USA). The shells were laser-scribed at the blunt pole along a round path of 15 mm diameter. The shell window was gently lifted using a scalpel. The inner shell membrane was left intact. After measurement, embryonic tissue samples were isolated for subsequent molecular sexing, or the shell windows were closed using a biocompatible adhesive tape (Leukosilk, BNS Medical GmbH, Hamburg, Germany) and further incubated until hatching. Visual assessment of health status and sex was performed on hatched chicks. LSL day-old chicks are sexable by discrimination of wing feather growth. In female LSL chicks primary wing feathers are longer than the covert feathers (“fast feathering”), while in male chicks primary and covert feathers show the same length (“slow feathering”) at hatch.

Reference sexing was obtained with genetic analysis on embryonic tissue based on polymerase chain reaction (PCR), as described elsewhere [13].

### Optical coherence tomography

A spectral domain optical coherence tomography (OCT) system [19] with center wavelength of 880 nm and bandwidth of 130 nm was used with an A-scan rate of 12 kHz. The system allows the simultaneous acquisition of 3D OCT and microscopy camera images by using the same beam path. For OCT, the near-infrared light from a superluminescent diode (Superlum, Russia) is guided to the scanner head containing a Michelson interferometer. The incoming light is separated by a beam splitter into a sample beam and a reference beam. The sample beam is rastered in x and y directions over the sample by two galvanometer scanners (Cambridge Technology Inc., USA). The backscattered and reflected light from different sample depth is guided back to the beam splitter and superimposed with the reference beam. This interference signal is spectrally resolved by a diffraction grating and measured by a line-detector (Teledyne DALSA Inc., Canada). The depth-dependent information is calculated by Fast Fourier Transformation and the logarithm of the intensity is displayed on an 8 bit gray scale. To acquire a 2D image of a sample, one galvanometer scanner is rotated to measure adjacent A-scans resulting in cross-sectional images. By deflecting the second galvanometer scanner, adjacent B-scans are acquired forming the 3D OCT image. The axial and lateral resolution is 6.5 μm in air.

### Optical spectroscopy

In ovo optical spectroscopy was performed with a spectrometer RamanRxn (Kaiser Optical Systems Inc., Ann Arbor, USA). The excitation was obtained with a diode laser emitting at a wavelength of 785 nm (Invictus 785-nm NIR, Kaiser Optical Systems Inc., Ann Arbor, USA). A fiber optic probe (MR-Probe-785, Kaiser Optical Systems Inc., Ann Arbor, USA) was used in the experiments. The excitation fiber had a core diameter of 62.5 μm and the collection fiber of 100 μm. The fiber probe was coupled to a self-built microscopy system enabling coaxial vision. Further details on the system are reported elsewhere [13]. A microscope objective 20×/0.4NA Plan Apo NIR (Mitutoyo Corp., Kanagawa, Japan) was used to focalize the laser beam in a spot with a diameter of ~ 55 μm. The measured laser power in the focus was 160 mW. The backscattered light was collected in reflection mode. In order to maximize the visibility of perfused blood vessels, illumination with green LEDs was employed. Blood vessels with diameters larger than 100 μm were manually chosen for the measurement. Total acquisition time was set to 40 s (20 accumulations of 2 s). The acquired spectral range was 150–3250 cm^-1^ and the spectral resolution was approximately 4 cm^-1^.

Scattering spectra of isolated native inner shell membranes were acquired with a Fourier-transform infrared spectrometer Vertex 70 (Bruker GmbH, Leipzig, Germany) equipped with a gold-coated integration sphere and InGaAs detector. The spectral resolution was 4 cm^-1^ in the spectral range 4000–15000 cm^-1^. The membranes were placed on a light trap, to avoid background reflection. Spectra were acquired using 200 scans.

### Data analyses

OCT datacubes were visualized and analyzed in Fiji [20]. The stack reslice function was used to generate cross sections and top views along user defined lines.

Spectroscopic data were analyzed using the Matlab package (MathWorks Inc., Natick, USA). The intensity of the fluorescence was calculated as sum area under the spectra in the range 500–2800 cm^-1^. Principal component analysis (PCA) was performed on the raw spectra by using the Matlab function “princomp”. The classification of spectra was carried out by an in-house written algorithm described elsewhere [12]. Briefly, a combination of two algorithms was employed to seek features to partition the spectra cleanly into two groups according to the sex. The first algorithm is a genetic optimal region selection routine. This algorithm takes as input the so called training spectra (in the present case, 40 spectra for each class) and their designation to female or male, respectively. With this information, the algorithm identifies a set of spectral subregions that, taken together, serve as a basis to group the spectra according to the sex. The classification was then carried out using a second algorithm which employs non-linear discriminant analysis to optimally partition into groups the spectra re-expressed as spectral subregions.

Statistics were calculated with Prism 6.0 (Graph Pad Software Inc., La Jolla, CA, USA).

## Results

**Fig 1:**
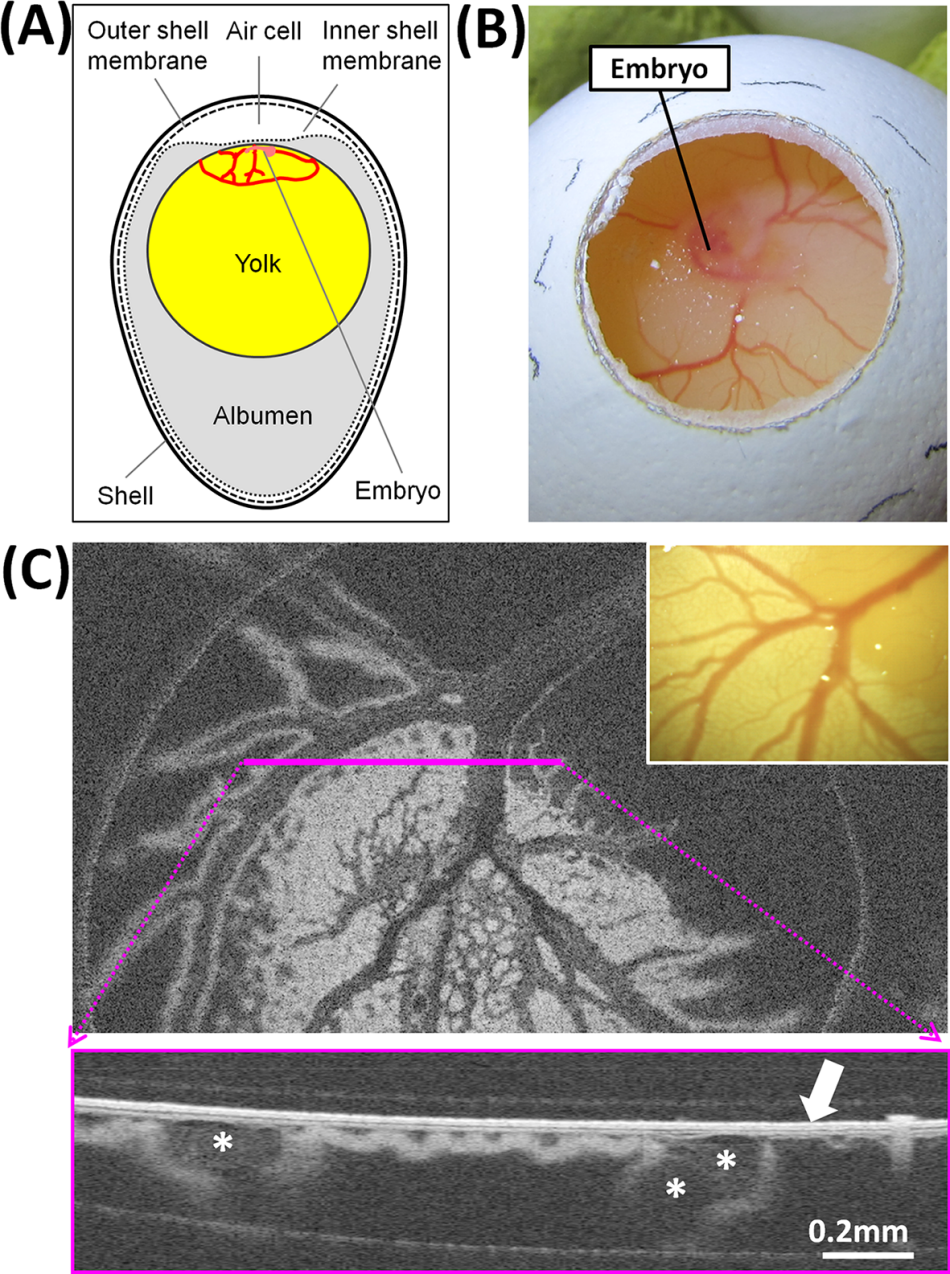
Structure of a chicken egg at day 3.5 of incubation. **A:** schema of the structures. **B:** embryo and extraembryonic vessels made accessible by opening the eggshell at the blunt pole. **C:** OCT image of extraembryonic vessels with corresponding camera image shown in the inset; in the cross-section OCT image acquired along the pink line, the arrow indicates the inner shell membrane and the asterisks indicate large blood vessels.

At day 3.5 of incubation of the chicken egg, the embryo is about 5 mm long and possesses a primitive blood circulation enabling gas exchange trough an extraembryonic vascularized area of the yolk sac. This vascularized area is referred to as vitelline circulation and has a diameter of about 3 cm. When the egg is in an upright position with the blunt pole facing upwards, the embryo in the center of the vascularized area is localized on top of the yolk, below the air cell at the blunt pole. The air cell is localized between the inner shell membrane, which is directly in contact with the albumen, and the outer shell membrane, which is adherent to the shell (Fig 1A). When a window in the shell is produced at the blunt pole, the outer membrane is removed together with the shell, while the inner membrane remains undamaged. The inner membrane is so thin that the embryo with the vitelline circulation is visible through the membrane (Fig 1B), thereby enabling identification of the vessels under a Raman microscope, the irradiation of the circulating blood with a near infrared laser and the acquisition of the backscattered spectrum.

The morphology of egg structures beneath the membrane, and relevant for the optical spectroscopic measurement, was studied by OCT and is exemplarily shown in Fig 1C. In the OCT image plane about 100 μm below the surface, the blood vessel network is clearly visible. In the cross-section OCT image, the inner shell membrane is visible as a bright structure on the surface. The blood vessels are localized immediately under the membrane. The thickness of the inner shell membrane measured on OCT images of 20 eggs was 22 ± 3 μm (mean ± SD, min. 18 μm, max. 27 μm). This is in substantial agreement with the reported value of 20 μm [21].

**Fig 2:**
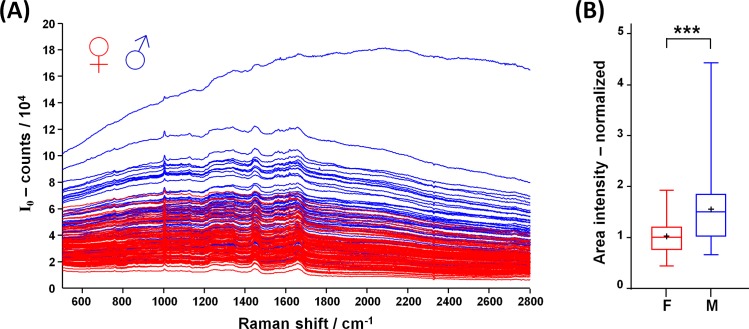
Spectra acquired in ovo through the intact inner shell membrane. **A:** spectra acquired on perfused blood vessels at the blunt pole of female (red) and male eggs (blue). **B:** Fluorescence intensity (line: median, cross: mean, box: 25^th^ to 75^th^ percentiles, whiskers: min-max; Mann-Whitney test, *** p < 0.001).

The spectra excited with near infrared laser radiation and acquired in ovo from blood vessels are composed by Raman bands superimposed to a broad fluorescence. As shown in Fig 2A, the fluorescence intensity of female eggs (n = 91) is generally lower compared to the fluorescence intensity of male eggs (n = 68). Median intensity values are significantly different, although a large overlap of fluorescence intensities exists, as shown in Fig 2B (data available in S1 Table). This result is similar to what we reported in former studies performed on the pointed pole using the same excitation and acquisition parameters [13]. However, due to scattering effects on the membrane, the intensity is reduced compared to the analogous experiments performed at the pointed pole.

**Fig 3:**
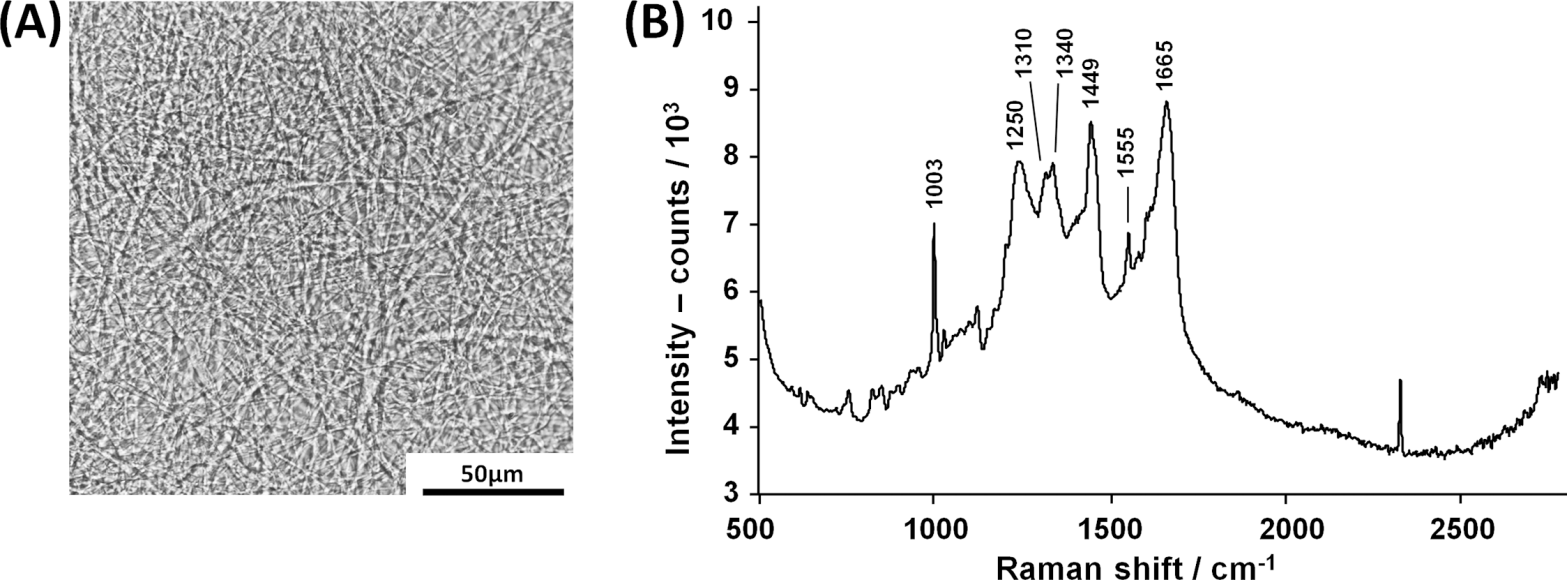
Inner shell membrane. **A:** Bright field microscopy picture. **B:** Raman spectrum of inner shell membrane, acquired on a native sample, freshly isolated from an egg.

The inner shell membrane is an optical inhomogeneous scattering medium due to its fibrous structure (Fig 3A) and possesses Raman-active molecular transitions. The spectrum of an isolated inner membrane is shown on Fig 3B. It mainly contains Raman bands related to proteins: aromatic ring vibration of phenylalanine and tryptophan at 1003 cm^-1^, amide III at 1250 cm^-1^, CH_x_ vibrations of aliphatic side chains at 1310, 1340 and 1449 cm^-1^, C = C vibration (likely of tryptophan) at 1555 cm^-1^, and amide I at 1665 cm^-1^ (all assignments based on [22,23]).

**Fig 4:**
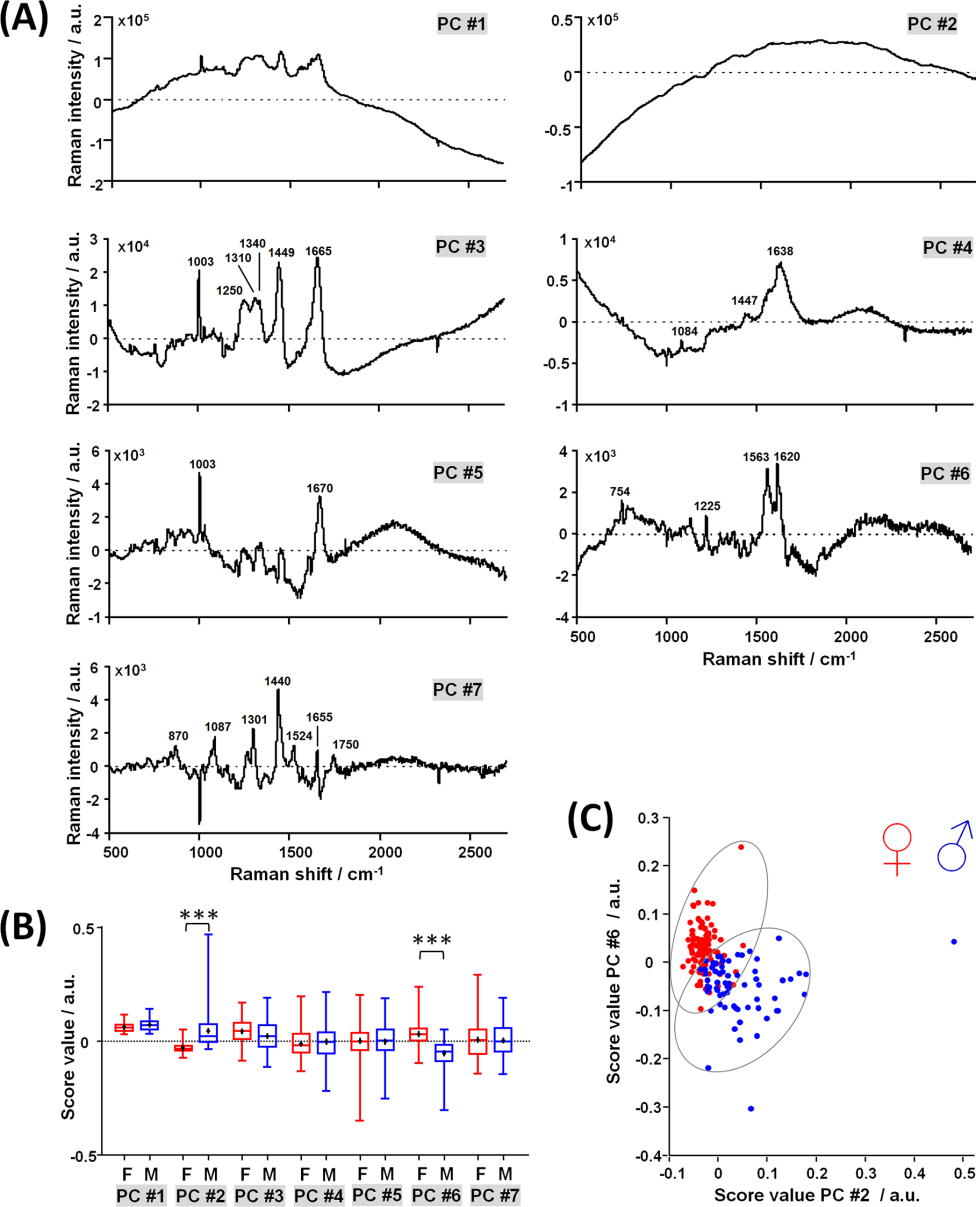
Principal component analysis of spectra acquired on perfused blood vessels at the egg blunt pole. **A:** Loading vectors; the position of Raman bands discussed in the text is indicated. **B:** Scores (line: median, cross: mean, box: 25^th^ to 75^th^ percentiles, whiskers: min-max; Mann-Whitney test, *** p < 0.001). **C:** Scatter plot of PC #6 vs. PC #2 scores.

PCA was applied on the spectra acquired in ovo on perfused blood vessels in order to gain better insights in the different spectral contributions of blood and membrane, and highlight the relationship with the sex. The loading vectors and the scores of the first seven principal components (PC) are shown in Fig 4 and given in S2 Table. Higher components contain no meaningful bands and represent just noise.

PC #1 describes the mean spectrum. PC #2 represents a fluorescence centered at ~ 1800 cm^-1^ (~ 910 nm). PC #3 is characterized by Raman bands of proteins as seen in the spectrum of the membrane, to which it is very similar. PC #4 contains bands at 1084 cm^-1^, 1447 cm^-1^ and 1638 cm^-1^, which can be attributed to C-N, CH_x_ vibrations and amide I of alpha helix secondary structure, respectively. PC #5 displays mainly the phenylalanine band at 1003 cm^-1^ and the amide I band of beta sheet secondary structure at 1670 cm^-1^. PC #6 contains bands at 754 cm^-1^, 1563 cm^-1^ and 1620 cm^-1^ likely attributed to porphyrin, and 1225 cm^-1^ attributed to amide III. PC #7 contains bands attributed to different molecular vibrations of lipids and phospholipids: 870 cm^-1^ attributed to antisymmetric stretch vibration of choline group N^+^(CH_3_)_3_), 1087 cm^-1^ attributed to C-C and PO_4_^3-^ stretching vibrations, 1301 cm^-1^ attributed to CH deformation, 1440 cm^-1^ attributed to CH_2_ stretching vibration, 1524 cm^-1^ and 1655 cm^-1^ attributed to C = C stretching vibrations, 1750 cm^-1^ attributed to C = O stretching vibration. All assignments were based on [22,23].

PC #2 and #6 describe variances that are significantly different between sexes (Mann-Whitney test, p < 0.001), supporting the idea that sex-related information are indeed contained in the spectra. However, data overlap is too large to enable separation between sexes with high accuracy (Fig 4C). PC #3 likely represents the contribution of the membrane. As it constitutes the third highest source of variance in the data, it suggests a strongly variable effect of the membrane on the acquired spectra, also in line with the variable membrane thickness measured with OCT.

**Fig 5:**
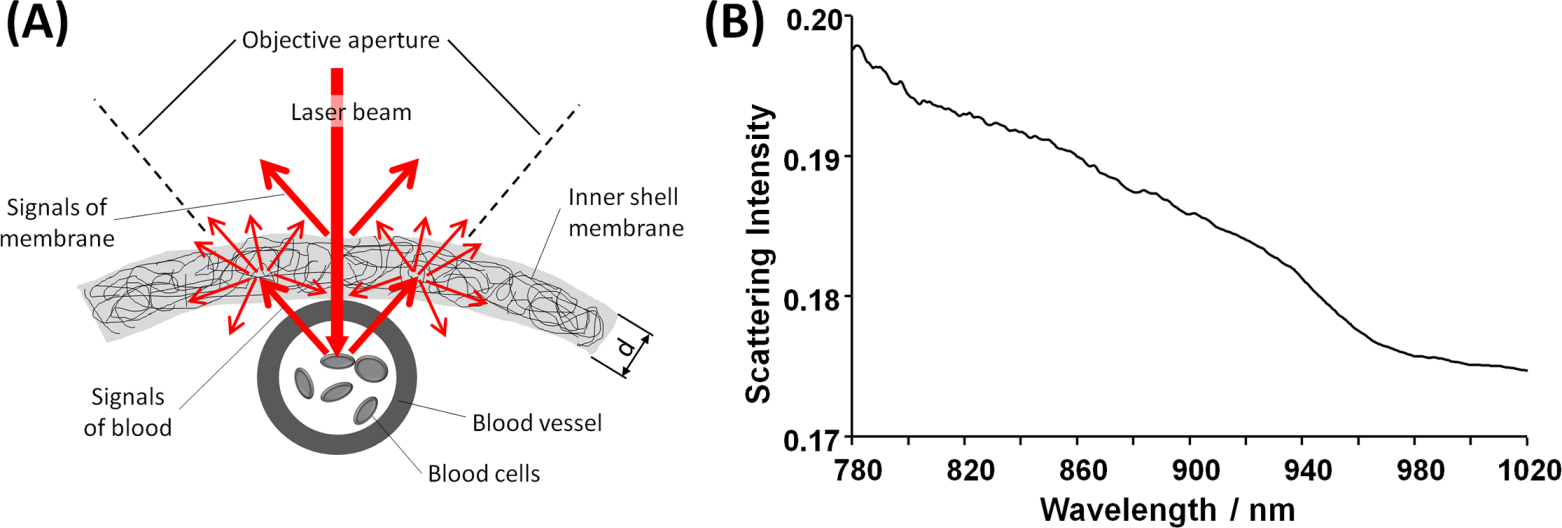
Scattering of the inner shell membrane. **A:** Schematic of the scattering effects. **B:** Scattering intensity of the inner shell membrane.

The presence of the inner shell membrane has a double effect on the excited fluorescence and Raman signals of blood cells, which will be (i) scattered by the membrane leading to a reduced measured intensity and (ii) superimposed by Raman bands of the membrane, as depicted in Fig 5A. Moreover, the excitation laser beam is also partly backscattered by the membrane, contributing to decrease the Raman and fluorescence signal intensities of blood.

In order to ensure high accuracy of classification, the impact of the inner shell membrane on the spectra of blood cells has to be compensated. For instance, the variability of membrane thickness introduces scattering losses that variably affect the blood spectrum of each egg.

As shown in Fig 3B, a dominant band in the Raman spectra of the membrane is located at 1003 cm^-1^, which originates from the aromatic amino acids phenylalanine and tryptophan. These amino acids are, beside glycine and proline, the most prevalent components in the inner egg shell membrane [24]. Furthermore, the results of PCA confirm that the intensity of this band is not related to the sex (i.e., it is not contained in the loading vectors of PC #2 and #6). Therefore, the intensity of this band in the spectra acquired on perfused blood vessels carries information on the thickness of the membrane. In order to exclude the background, a two-point linear baseline correction was performed on the spectra acquired on perfused blood vessels and on the spectrum of the membrane in the range 980–1020 cm^-1^, resulting in the spectra *S*_*BL*_ and *Sm*_*BL*_, respectively. The normalized thickness of the membrane *d’* was calculated as ratio of integral band intensities:

$$d^{\prime }=\frac{\int_{980}^{1020}S_{BL}(\nu )\;d\nu }{\int_{980}^{1020}S_{mBL}(\nu )\;d\nu }$$ (1)

In order to compensate for Raman signals of the inner shell membrane, the spectra acquired in ovo were corrected as follows:

$$I\prime =(I_{0}-S_{R}d^{\prime })$$ (2)

where *S*_*R*_ is the Raman spectrum of the membrane (see Fig 3B) and *I*_*0*_ the spectrum of embryonic blood.

A simple approach to compensate the scattering of the membrane is based on the theory of Kubelka and Munk [25]. As the absorption of the membrane is very low in the spectral range from 500 to 2800 cm^-1^ Raman shift (approx. 815 to 1005 nm), the theory can be simplified and the transmission expressed as [26]:

$$T=\frac{1}{1+Sd}$$ (3)

where *S* is the scattering coefficient and *d* the thickness. Eq (3) provides an estimate for the relative changes of transmission losses *T'* due to scattering on membranes with different thickness, which is given by:

$$T\prime (\lambda )=\frac{1}{1+S_{m}(\lambda )d^{\prime }}$$ (4)

*S*_*m*_*(λ)* is the scattering intensity, which was measured on the freshly isolated membranes and is shown in Fig 5B in the spectral range of interest. Finally, the corrected spectral intensity *I*_*c*_ was retrieved as:

$$I_{c}=I^{\prime }∙\frac{1}{T\prime (785)}∙\frac{1}{T\prime (\lambda )}$$ (5)

The term *T'(785)* was obtained from Eq 4 using the scattering intensity *S*_*m*_ at 785 nm and compensates the relative variations due to scattering of the excitation beam, while the term *T'(λ)* compensates for the wavelength-dependent relative variations due to scattering of the blood signals.

**Fig 6:**
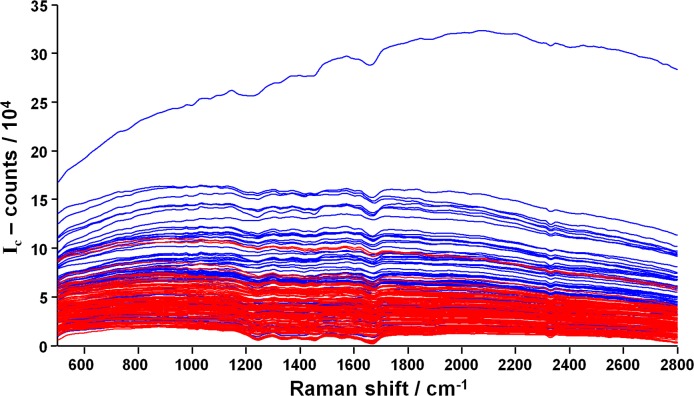
Correction to compensate for inner shell membrane effects. Spectra acquired on perfused blood vessels at the blunt pole of female (red) and male eggs (blue), after the correction described by Eq 5.

The corrected spectra are shown in Fig 6. The ratio between male and female median intensities is 2.3, which is higher than the ratio of 1.6 calculated from the raw data.

**Fig 7:**
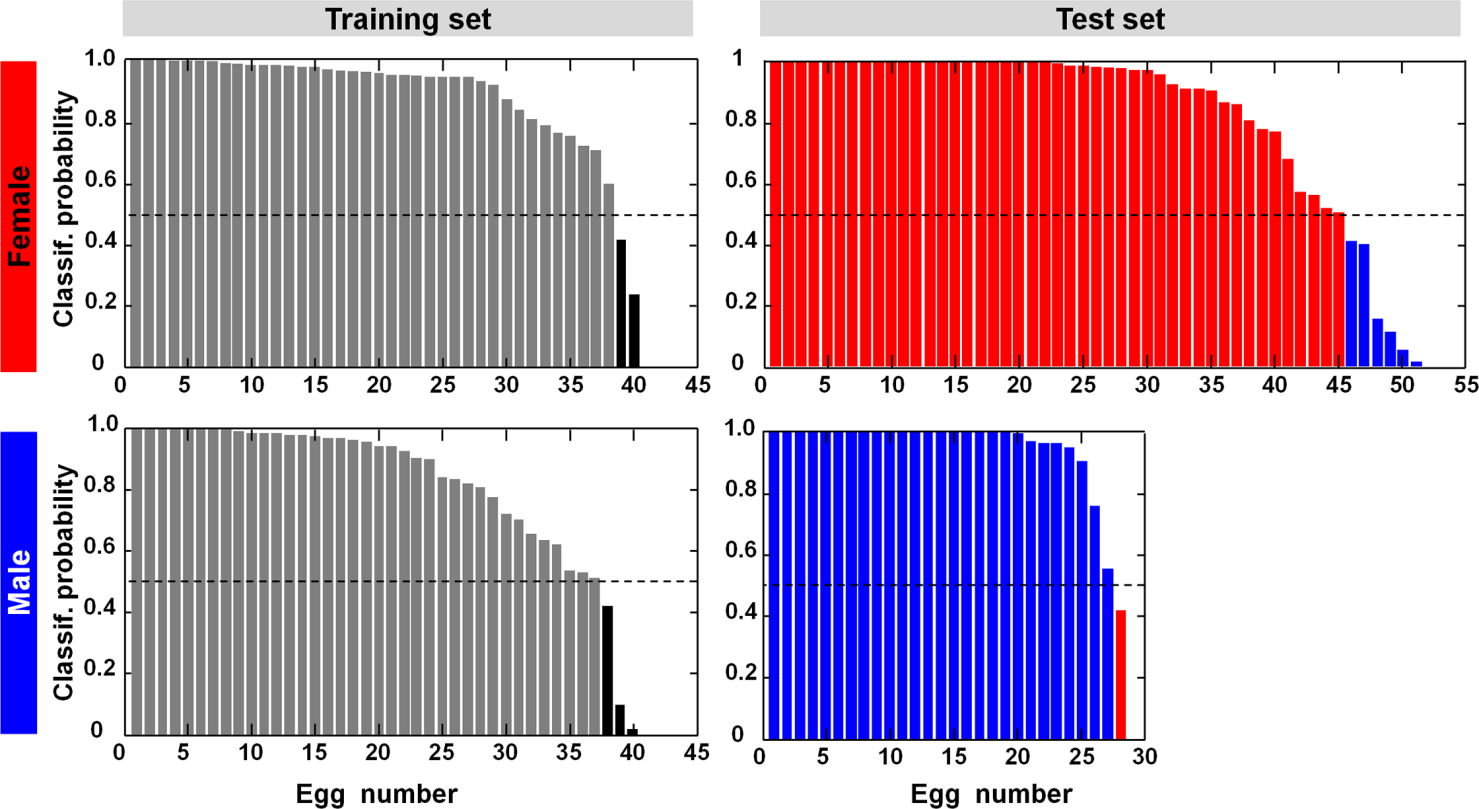
Supervised classification for egg sexing. The probability of class membership is reported for each spectrum of the training set and the independent test set of both classes.

Finally, the corrected spectra were classified to retrieve the sex. A supervised classification was trained on 80 corrected spectra that were randomly selected (40 females and 40 males), and afterwards applied on the remaining 79 corrected spectra (51 females and 28 males), which were used as independent test set. The classification approach was based on spectral feature selection and iterative classification optimization. The selected spectral features were 550, 715, 912, 1215, 1304, 1958, 1966, 2214 cm^-1^. The classification algorithm of nonlinear discriminant analysis supplied the probability of membership to female and male for each spectrum, which is shown in Fig 7. The reclassification of the training set was performed with a correct rate of 94% (females: 38/40, males: 37/40). The spectra of the test set were classified with a correct rate of 91% (females: 45/51, males: 27/28).

Hatching experiments were performed with 71 eggs that were randomly selected after optical spectroscopy measurement. The egg shell windows were closed with medical-grade gas-permeable adhesive tape, and the incubation continued under standard conditions of temperature and humidity. Further embryonic development and hatching behavior, as described elsewhere [27], were not affected. Healthy chicks hatched from 96% of eggs (68/71), without differences compared to intact eggs. All three embryos died at around day 19 (stage 45 [28], after the transfer from the incubator into the hatcher. The visual inspection of dead embryos did not provide any indication for a bacterial or fungal infection, or for malformations. A slightly retarded development was observed in two of the dead embryos, because the yolk sac was not fully absorbed. For the domestic chicken, it is well investigated that there are two time periods within the incubation where mortality of embryos is high, i.e., between the third and fifth days and on approximately the 19^th^ day. Artificially incubated embryos are much more likely to die during the later period of susceptibility than embryos incubated under the hen. In the late critical period embryos might die because of failure to make a proper transition from the allantoic to pulmonary respiration, poor development of the hatching muscle, deficient oxygenation, water starvation, or a cumulative effect of all unfavorable conditions [29,30]. Hatched chicks did not display physical or behavioral anomalies. All chicks were clean with shiny dry down feathers without persisting feather sheets. Their eyes were clear and bright. Legs, feet and toes were straight without any deformities. The belly of each chick was soft and showed a clean dry navel without swelling. All chicks displayed exploratory behavior and started intake of food and water within a short time after hatching.

## Discussion and conclusions

Sexing of circulating embryonic blood by optical spectroscopy is possible based on information carried by near infrared fluorescence and Raman signals. Fluorescence intensity of male blood is significantly higher compared to females. PCA also reveals a different spectral shape of fluorescence: the blood of males is characterized by a fluorescence band localized at about 910 nm. Sex-related differences are also present in the Raman spectrum, and PCA indicates differences in blood protein content. This is in agreement with the results of measurements performed at the pointed pole.

The presence of the inner shell membrane above the vessels affects the signal of blood in several ways. The membrane is a thin layer of proteinaceous fibers. First, it introduces Rayleigh scattering, inducing a decrease of signal intensity that depends on membrane thickness. Moreover, Raman bands of the membrane overlap with the spectrum of blood.

For these reasons, reliable sexing needs proper correction strategies of the spectra. The presented approach permitted to compensate for variable signal losses due to Rayleigh scattering caused by membranes of different thicknesses, as well as subtract their Raman contributions. After correction of the spectra, the feature selection algorithm identified spectral regions that mainly describe the fluorescence. Egg sexing based on supervised classification of the spectral features attained an overall correct rate of 93% (147/158). This result is fully comparable with in ovo spectroscopic sexing performed at the egg pointed pole without shell membrane [12,13].

The optical spectroscopic measurement on perfused blood vessels at the egg blunt pole enables to overcome several drawbacks of similar measurements performed at the pointed pole, where shell windowing implies also removal of eggshell membranes. The presence of the air cell at the blunt pole allows for shell fenestration while leaving the inner shell membrane intact. As this membrane provides main protection of the embryo against external bacteria, shell windowing at the blunt pole does not impact negatively on hatching rate. In contrast, egg fenestration at the pointed pole with a window of 15 mm in diameter led to a hatching rate reduction of 11% compared to control eggs without shell window (81% vs. 92%) [12].

The method here illustrated satisfies all requirements for in ovo sexing [31]. By leaving the inner membrane intact, the egg environment remains protected against external influences, which avoids any reduction of hatching rates. As also proven in former experiments, the optical measurement is “per se” damage-free, and does not have any negative effects beyond the ones related to shell windowing. Post-hatch animal behavior and development are not affected as well. From a technical point of view, the technique offers the possibility of full automatization and does not require consumables, which is economically important for sexing of large amounts of eggs. Sexing is performed in seconds, so that male eggs are sorted out immediately after measurement. These could be used as protein source depending on national regulations, for example for fish feeding. Finally, the method improves animal welfare, as it is conducted before development of embryo sensitivity, and is therefore ethically more acceptable than culling of day-old chicks.

Compared to optical spectroscopic sexing performed at the pointed pole, the measurement at the blunt pole simplifies process automatization. As eggs are incubated with the blunt pole upward, shell windowing and measurement at the blunt pole provide several advantages. Handling is reduced, as turning of eggs before and after the measurement is no more required. Shell windowing is facilitated by presence of the air cell, which creates a gap between shell and egg structures. Damage-free laser windowing can thus be performed on the incubated eggs immediately before measurement. Moreover, the procedure of shell window sealing in female eggs after sexing is no more critical, as the risk of egg fluids’ loss is avoided by upright positioning during incubation.

In conclusion, spectroscopic sexing at the blunt pole qualifies as a minimally invasive, precise method that facilitates egg handling and process automatization, thereby offering the best premises for deployment in the layer industry.

## Supporting information

S1 Table Fluorescence intensity of female eggs (n = 91) and of male eggs (n = 68), used to produce Fig 2B. (PDF)

S2 Table Scores of principal component analysis (female eggs n = 91, male eggs n = 68), used to produce Fig 4B and 4C. (PDF)
