# Supplementary material for: Sexing of chicken eggs by fluorescence and Raman spectroscopy through the shell membrane
Source: PLoS One. 2018 Feb 23;13(2):e0192554. doi: 10.1371/journal.pone.0192554 (PMC5824995; doi:10.1371/journal.pone.0192554)
Supplement: S1 Table — (PDF) [file pone.0192554.s001.pdf]

Sexing of chicken eggs by fluorescence and Raman spectroscopy through the shell membrane

Roberta Galli, Grit Preusse, Christian Schnabel, Thomas Bartels, Kerstin Cramer, Maria-Elisabeth Krautwald-Junghanns, Edmund Koch, Gerald Steiner

S1 Table: Fluorescence intensity of female eggs (n = 91) and of male eggs (n = 68), used to produce Fig 2B.

Female	Male
59465740	170213300
44659410	137423100
118887200	78493810
95654380	168850800
85316700	120435300
89867580	149403300
53714480	152990900
60386700	154619100
105383000	165146800
111704000	124499700
103018900	65906750
63105720	96026340
99364510	176001900
86546120	103286000
75963280	121070700
72534610	95718460
106569600	160533900
96022490	182550000
156644300	106273000
65999200	101333500
124742100	155613000
67684890	67139420
67043550	93872300
81247970	138203700
137515300	184952800
69263780	91947230
63344750	82798700
110782600	303658700
79611540	90834750
87956700	130907700
85247670	158917800
74216660	138995000
119022800	79439380
101318000	74977690
166579900	198008400
118829100	70805460
82446310	169149900
130630500	85404270
66702540	75408630
116944600	147241700
103098200	256487700
103207000	190257800
75193700	139052500
83311850	120895900
118414300	434952400
99480190	229123300
177281300	228025400
120718700	193036700
108607500	170047800
144788200	216172300
84603180	247789700
62306210	178690000
117904200	179304200
115285500	226756900
75809780	239383100
144394500	240129900
87722790	118847400
157699200	106957400
79510880	158160800
148701600	91716980
111938900	90161660
94304020	133594100
147216900	176853900
189883000	122375200
93265470	108675000
126086000	160277100
85233880	251078200
71922740	237614000
128033100	
168025600	
105617700	
86032290	
132884600	
67488900	
106298800	
101201200	
69201760	
120103400	
80775190	
104779400	
110506900	
150772500	
82238100	
116095800	
98197380	
63262100	
142743100	
58442340	
108709700	
110426500	
71926060	
