# Supplementary material for: Sexing of chicken eggs by fluorescence and Raman spectroscopy through the shell membrane
Source: PLoS One. 2018 Feb 23;13(2):e0192554. doi: 10.1371/journal.pone.0192554 (PMC5824995; doi:10.1371/journal.pone.0192554)
Supplement: S2 Table — (PDF) [file pone.0192554.s002.pdf]

Sexing of chicken eggs by fluorescence and Raman spectroscopy through the shell membrane

Roberta Galli, Grit Preusse, Christian Schnabel, Thomas Bartels, Kerstin Cramer, Maria-Elisabeth Krautwald-Junghanns, Edmund Koch, Gerald Steiner

S2 Table: Scores of principal component analysis (female eggs n = 91, male eggs n = 68), used to produce Figs 4B and 4C.

FEMALE						
**PC#1**	**PC#2**	**PC#3**	**PC#4**	**PC#5**	**PC#6**	**PC#7**
0.039998	-0.03342375	0.05429354	-0.04993797	0.1173505	0.00379161	0.189611
0.03023673	-0.03383821	0.0479674	-0.06572527	0.08796153	0.08293096	0.05374194
0.07505866	-0.04145262	0.05301946	0.03724666	0.1328884	0.1236001	-0.1087836
0.04202347	0.03036664	0.1694113	0.07136149	0.2044175	-0.04616725	-0.08231712
0.05423988	-0.04007672	0.02669309	-0.04538325	0.1010646	0.08856021	-0.05602665
0.04997856	0.04612415	0.05230108	-0.02359084	-0.3509687	0.2386086	-0.1353566
0.03571101	-0.02932984	0.0833483	-0.00290222	0.05492726	0.04436014	0.08374703
0.03795726	-0.0195509	0.04039111	-0.03095433	0.01267002	0.04689846	0.0987814
0.05057935	0.0353656	0.06005153	0.03117827	0.02990309	-0.01979186	-0.05392768
0.07245508	-0.05035544	-0.01167689	-0.06674655	0.03699273	0.1173178	-0.05808302
0.06650005	-0.04651166	0.05272357	-0.08542729	0.06492173	0.0350484	-0.08716863
0.04350348	-0.03425311	0.1085761	-0.01376377	-0.03152838	-0.01084209	-0.00824744
0.03428776	0.05038149	0.05818152	-0.06550259	-0.01832683	0.0349654	-0.05889725
0.05236962	-0.01570484	0.05757273	-0.05567165	-0.1007892	0.00564997	0.09448263
0.05148713	-0.03837736	0.1084674	0.05440446	0.05108324	0.03990537	-0.06240959
0.04240297	-0.02149607	0.09158091	0.00739776	0.07105593	0.03589754	-0.0384216
0.05565573	-0.01126678	0.00263815	-0.01179619	0.0700015	0.1211052	0.04043057
0.05334679	0.00449892	0.03360611	-0.02503716	-0.08890167	0.04687369	-0.06154155
0.09216767	-0.02294659	-0.02114091	0.09377745	0.03552388	0.1240046	0.1996016
0.04456947	-0.03472999	0.09346288	0.01699409	0.0259035	0.04051316	0.04417278
0.07046613	-0.00719563	0.03309239	0.04236834	0.03129327	0.02339217	0.0213992
0.04604933	-0.04674553	0.1614336	0.03284572	0.1523726	0.00333804	-0.09185059
0.0399769	-0.03065287	0.05576394	-0.08127473	-0.00905612	0.04207151	0.0752439
0.04636955	-0.01812564	-0.01122741	-0.1023648	0.01610911	0.01685224	-0.00184836
0.08223316	-0.03647838	-0.01316384	0.0218586	0.03416166	0.07285002	0.05129099
0.04085669	-0.02835854	0.0164281	-0.09544767	-0.01265314	0.07031172	0.03838567
0.04479096	-0.03155501	0.07559485	-0.0133176	-0.08555722	-0.02171179	0.08978136
0.07388086	-0.03030824	0.1227401	0.123726	-0.0439691	-0.02419286	-0.081644
0.04314033	-0.00743032	0.03665175	-0.04667463	-0.02992078	0.06583299	0.04734675
0.04729903	0.00303375	0.08584443	-0.02076882	-0.05617735	-0.02560542	-0.02460709
0.05626596	-0.04137359	0.1232907	0.04804921	0.01914056	0.01549815	-0.01236743
0.04581929	-0.01829673	0.07552465	0.01132353	-0.08112697	0.0242315	0.07543023
0.07693418	-0.03810001	0.05585644	-0.01667295	-0.00290574	0.01978459	0.00270459
0.06778496	-0.0529	0.04845666	-0.04567295	0.0020975	0.05682761	-0.07371379
0.1025973	-0.04907801	0.00166437	0.04697407	0.08411908	0.1485426	-0.06390268

0.06726384	-0.00932195	0.06383127	-0.03595135	-0.02243624	0.05248906	-0.1092064
0.05448051	-0.03792736	0.1244627	-0.04592538	-0.03489828	-0.02103335	0.04142047
0.07542444	-0.00111907	0.1631434	0.1648646	-0.03798982	-0.04926275	0.1017453
0.0415432	-0.03326083	0.08124453	-0.02773575	0.02490416	0.02199061	-0.0286205
0.0709052	-0.0338405	0.01594236	-0.02382242	0.03565115	0.04786957	-0.0623121
0.07117614	-0.05794146	0.08043002	0.02126416	0.0279034	0.03211077	-0.1163887
0.06361352	-0.02136544	0.03360401	0.04932851	-0.06820183	0.06006607	0.04114309
0.04287503	-0.02284541	0.01314508	-0.08389752	-0.00350543	0.06029913	0.01920969
0.04557557	-0.02276177	0.00225536	-0.04763384	0.04434826	0.0914081	0.07054801
0.07406206	-0.02765474	0.00820525	0.03338312	-0.06255632	0.03181453	0.03832171
0.0626667	-0.03216063	0.0949628	-0.00268976	-0.0175506	0.01361905	0.06766099
0.1034379	-0.01476367	-0.08512393	0.03071059	0.03009583	0.07806197	-0.02854012
0.07877814	-0.02376232	0.05413022	-0.00967692	-0.1341181	0.04295342	-0.128639
0.05737828	0.00554727	0.09111609	0.00479904	-0.04199518	-0.00182655	0.0499839
0.08914255	-0.02130993	-0.02028758	-0.04269204	-0.04475302	0.0566984	-0.09213305
0.04721271	-0.00158876	0.02100482	-0.02948539	-0.1179353	0.02030132	-0.00593608
0.03779797	-0.02737891	0.02484796	-0.09675947	-0.0111245	0.03071787	0.00721691
0.07351199	-0.02670568	0.02481415	-0.01464545	0.09824479	-0.05548673	-0.01721152
0.07206899	-0.02600828	0.00830742	-0.03056691	-0.05112575	0.04737268	-0.00900591
0.05358257	-0.04223654	0.08484603	-0.001294	-0.00101375	0.01558485	0.06781496
0.08304498	-0.03879496	0.00933537	-0.03223018	0.1252339	0.03076476	0.06885148
0.05715833	-0.06152023	0.02503232	-0.0733811	-0.01818371	0.04755087	0.2930166
0.09681927	-0.05201612	0.03684699	0.06166172	0.07610444	0.1188874	-0.01652706
0.04998377	-0.05165116	0.00571731	-0.1333077	0.06988242	0.03892955	-0.03926391
0.09367355	-0.04207169	0.01835625	0.02681642	0.03497598	0.04854506	-0.04904267
0.06987132	-0.01798737	-0.01603412	-0.03721779	-0.08182376	-0.06209322	0.06135299
0.06183079	-0.05782092	0.0058482	-0.09255561	-0.01690287	0.08265191	-0.00467848
0.0882271	-0.02759454	-0.01350899	-0.0008695	-0.02241549	0.02807355	0.02072682
0.1169973	-0.04172101	-0.07167827	0.06427167	0.09651341	0.0899638	0.02301401
0.05917099	-0.07242732	-0.01787278	-0.1133635	0.1015081	-0.00928224	0.07613411
0.07876661	-0.03261179	0.03131624	0.03671709	-0.02879972	0.0801509	-0.02346493
0.05139347	-0.0535112	0.00328814	-0.126439	0.03823071	0.06590542	0.02106697
0.04677151	-0.03923531	0.08020684	0.03332239	0.00464551	0.01885399	0.08559078
0.08210402	-0.04559799	0.01311823	0.03988344	0.01693773	0.0489285	0.00459785
0.1057986	-0.03423186	-0.02568261	0.0945266	-0.02569396	0.04393408	0.1458647
0.0648916	-0.06183337	0.00649001	-0.06506654	0.02070112	0.00317688	0.04743376
0.05945204	-0.05129975	0.01546274	-0.0203273	-0.02889412	0.00696876	0.03367272
0.08126886	-0.05242971	-0.01565428	-0.05976477	-0.00326321	0.04671578	0.03195003
0.04200975	-0.03546599	0.04801425	-0.06833676	-0.00842692	-0.00040401	0.03133021
0.06299705	-0.05319891	0.05134622	-0.01819798	0.09912033	0.01879201	-0.03421134
0.06590791	-0.05452167	0.1233899	-0.0108896	0.01342351	0.0264002	-0.1418076
0.04312974	-0.02680507	0.1113435	0.00391316	0.00771025	-0.00552246	-0.06226006
0.06334181	0.01150501	0.04748954	0.01167739	-0.05509232	0.01332658	0.01422223

0.04655324	-0.02453414	0.01805066	-0.05017363	-0.011264	0.05575463	0.0054385
0.05715742	-0.02239748	-0.02649877	-0.04049138	-0.0050561	0.01986653	0.03594993
0.0630478	-0.02259533	0.05017797	-0.09994454	-0.05459315	0.02024025	-0.1034377
0.09225013	-0.02322497	0.04495704	0.1952957	0.02545837	0.03710558	0.03429001
0.05664365	-0.04248392	0.1191855	0.04956712	-0.0356594	-0.04732344	0.06887976
0.0705367	-0.03657846	0.02580089	-0.05944141	-0.00206769	0.01753376	-0.03899684
0.06144481	-0.03808733	0.03605601	0.03287108	0.02423178	-0.01441989	-0.05717954
0.04931478	-0.03568602	0.1228672	0.07129279	-0.1031205	-0.09649809	-0.01083926
0.09902203	-0.05087767	-0.03050376	0.0729552	-0.05213637	-0.00935008	-0.01521187
0.04088362	-0.05492985	0.0782366	-0.08195345	0.01853722	-0.02014541	-0.06531245
0.06441738	-0.01903707	0.04494849	-0.02271248	-0.09071848	-0.02394812	0.01687964
0.06932499	-0.03711002	0.08591921	-0.03873574	-0.06431549	-0.04803766	0.00549319
0.04653785	-0.03651664	0.06627402	-0.03474091	-0.05431938	-0.03947439	0.05553567
**MALE**						
**PC#1**	**PC#2**	**PC#3**	**PC#4**	**PC#5**	**PC#6**	**PC#7**
0.07346832	0.07766338	-0.03690509	0.00537521	0.179842	-0.03487345	0.01669892
0.05903339	0.05325626	0.09440046	0.01698799	0.1894822	-0.04705044	-0.04955554
0.03630751	0.0208724	0.02016155	-0.05900478	0.03120591	0.00272789	0.03119228
0.07359931	0.08144721	0.06005386	0.00092679	0.0625685	-0.09506905	0.05705633
0.07109673	-0.0158733	0.03176162	-0.01891021	0.07416649	-0.0996599	-0.00151596
0.07393183	0.04021484	0.05872925	-0.02266252	0.09586658	-0.09820266	-0.1308994
0.04649766	0.115438	0.1063944	-0.04960067	-0.02813383	-0.07431281	-0.05431617
0.09278401	-0.02246359	0.03408069	-0.07135038	0.06629911	-0.06032663	-0.05835101
0.0842887	0.0353947	-0.02214658	-0.07088169	-0.03727351	-0.09462439	0.08388905
0.06568602	0.02268611	0.09926853	0.09218023	0.00583005	-0.04423497	0.09705626
0.0340136	0.00511638	0.04692358	-0.08940334	-0.02737242	0.01175781	0.0069329
0.05059748	0.00171625	0.09361126	-0.01349612	0.03491341	-0.02217379	-0.01696628
0.05206346	0.1649036	0.04487531	0.00413861	-0.1582397	-0.02188946	-0.04819117
0.05840709	0.00953091	0.1909735	0.1458115	0.04900286	-0.06575875	0.1349953
0.07067718	-0.0003141	0.00094205	-0.00393923	-0.04462774	0.01215768	-0.04194604
0.05030333	-0.00211171	0.0153904	-0.09966791	0.05907311	-0.04085391	0.1163519
0.0995184	-0.02192089	-0.02995689	-0.04418687	-0.0110847	-0.0498465	-0.07181635
0.09259985	0.04817661	-0.02375658	0.08228768	-0.04436462	0.01489207	0.1131529
0.0674073	-0.02591981	0.1044341	0.1242632	0.02412357	-0.0234505	-0.0116469
0.05121142	0.01830134	0.07396338	0.01136211	-0.03770083	-0.06258962	-0.00171659
0.06682125	0.0793948	-0.00970338	-0.02011839	0.00270277	-0.07619762	-0.04710947
0.04106454	-0.02561143	0.07299137	-0.0727448	-0.03702273	0.00237392	0.08471396
0.05899328	-0.02820604	0.1300951	0.04500138	0.06477365	-0.0149733	-0.1124049
0.04991369	0.08372589	0.0261433	-0.06055768	-0.03667925	-0.04706798	-0.01912499
0.09432766	0.04369193	-0.05189345	-0.133243	-0.0792005	-0.161186	-0.02460263
0.04397737	0.00908597	0.00526874	-0.0892578	-0.02908353	-0.02016512	-0.0014358
0.04617335	-0.00601417	0.0879263	-0.02426658	0.02322957	-0.0178715	-0.08915284
0.1357827	0.1237477	-0.06362899	0.203416	0.04997377	0.05047577	-0.04296237

0.04512624	-0.00457575	0.1099544	-0.09131783	0.04533331	-0.00256889	-0.04207561
0.07235972	0.01312309	0.03049104	-0.09986184	-0.1328748	-0.0936375	0.1178169
0.07446365	0.06067639	0.00979423	-0.00154703	-0.03060035	-0.09416044	-0.07674278
0.07395675	0.01970318	-0.00730105	0.00541433	-0.05742633	-0.02820208	-0.02802687
0.04355444	-0.00358935	0.1470373	0.0074085	0.00653446	-0.05320006	0.1280497
0.05010245	-0.02062528	0.04523043	0.04519319	-0.0646118	-0.05696474	0.06132761
0.07151285	0.1313329	-0.01982357	-0.00116159	-0.06573968	-0.03760587	0.01184658
0.03321417	0.00905475	0.05248976	-0.08400961	-0.0348556	0.00335884	0.1103347
0.08187406	0.07937657	0.01556202	-0.08034382	-0.03760049	-0.1521265	-0.1054367
0.04572837	-0.01029602	0.08456614	-0.03451285	-0.00037807	-0.00157233	0.07442932
0.04244977	-0.01114427	0.1013711	0.02141044	0.05465796	-0.0073976	-0.04542311
0.08257034	0.01837185	-0.00277399	0.0115677	-0.1013022	-0.04989106	0.00020117
0.1320545	0.1227203	-0.03741488	0.0724248	-0.2549678	-0.1009044	-0.1455114
0.07192884	0.06692021	-0.0788386	-0.2186991	0.07086429	-0.3032773	0.04985695
0.07724716	0.00724985	0.00051504	0.09642538	0.02004149	-0.00951775	0.07140925
0.06664909	-0.00237192	0.03785511	-0.04613815	-0.01573791	-0.05758406	-0.05457974
*-0.07673212**	0.4932952	*0.2346259**	*-0.1573403**	*0.1274392**	0.0437304	*0.1239451**
0.1139911	0.06430333	-0.09350127	0.07750636	-0.02413425	0.02335889	-0.04038006
0.1121488	0.07900671	-0.05588329	0.1176254	0.01908632	0.00730581	-0.07143132
0.1121972	0.03196979	-0.03841349	0.1061123	-0.03961632	-0.0912457	0.01186282
0.08270756	0.03798485	0.00989461	0.00479584	-0.03338294	-0.02662204	0.03963051
0.1032549	0.07472014	-0.09823155	0.03764557	0.06493215	-0.01862974	0.00785521
0.08176844	0.178992	-0.01688472	0.02589688	0.01289191	-0.02486729	-0.00518449
0.09770374	0.03349519	-0.07046333	-0.07547591	-0.05261742	-0.1377346	0.04877707
0.04937397	0.1475282	0.01530708	-0.01420226	-0.04993538	-0.03418599	0.05240617
0.1416908	-0.03579046	-0.112185	0.04267792	0.04025769	-0.01509912	0.00111333
0.08341931	0.1764771	0.02061465	0.1057242	-0.0695756	-0.06726508	-0.1033163
0.1312936	0.03932471	-0.1032221	0.2151524	0.00646112	0.01590471	0.06283113
0.06406943	-0.02258458	0.05324708	-0.01746807	0.05645326	-0.05511122	0.03184828
0.05465787	0.00312818	0.07068024	-0.02627587	-0.1026178	-0.06790934	0.00418823
0.08716922	-0.01291218	-0.00798896	0.01628433	0.0651567	-0.01026193	0.00427106
0.04106171	0.01481891	0.03539839	-0.05952333	-0.03841298	-0.0429772	0.07684065
0.05062107	-0.01822304	0.1001425	-0.00466972	0.01986667	-0.05258013	-0.05384792
0.06513016	0.02200885	-0.01779963	-0.00907942	-0.04029067	-0.05365205	0.06572208
0.06597462	0.09984177	0.05977351	-0.01304444	0.01028294	-0.1165957	-0.06051505
0.06873673	-0.02233615	0.1159738	0.05551024	0.11004	-0.03722055	-0.00910629
0.04383946	0.0443865	0.05440319	-0.02317278	-0.05013699	-0.1237755	-0.0254818
0.09088498	0.00361427	-0.03463318	-0.0007859	0.01122279	-0.08753941	-0.01365407
0.1404607	-0.02082452	-0.1136332	-0.01349087	0.06603864	-0.2177514	0.190835
0.09105778	0.120244	-0.04658403	-0.05245592	0.04241504	-0.1006857	0.00685233
